# Screening toll-like receptor markers to predict latent tuberculosis infection and subsequent tuberculosis disease in a Chinese population

**DOI:** 10.1186/s12881-015-0166-1

**Published:** 2015-04-01

**Authors:** Linlin Wu, Yi Hu, Dange Li, Weili Jiang, Biao Xu

**Affiliations:** Department of Epidemiology, School of Public Health, Fudan University, Shanghai, 200032 China; Key Laboratory of Public Health Safety (Fudan University), Ministry of Education, Shanghai, China

**Keywords:** Toll-like receptors, Polymorphism, Tuberculosis, Latent tuberculosis infection, Susceptibility

## Abstract

**Background:**

We investigated whether polymorphisms in the toll-like receptor genes or gene–gene interactions are associated with susceptibility to latent tuberculosis infection (LTBI) or subsequent pulmonary tuberculosis (PTB) in a Chinese population.

**Methods:**

Two matched case–control studies were undertaken. Previously reported polymorphisms in the toll-like receptors (TLRs) were compared between 422 healthy controls (HC) and 205 LTBI patients and between 205 LTBI patients and 109 PTB patients, to assess whether these polymorphisms and their interactions are associated with LTBI or PTB. A PCR-based restriction fragment length polymorphism analysis was used to detect genetic polymorphisms in the *TLR* genes. Nonparametric multifactor dimensionality reduction (MDR) was used to analyze the effects of interactions between complex disease genes and other genes or environmental factors.

**Results:**

Sixteen markers in *TLR1*, *TLR2*, *TLR4*, *TLR6*, *TLR8*, *TLR9*, and *TIRAP* were detected. In *TLR2*, the frequencies of the CC genotype (OR = 2.262; 95% CI: 1.433–3.570) and C allele (OR = 1.566; 95% CI: 1.223–1.900) in single-nucleotide polymorphism (SNP) rs3804100 were significantly higher in the LTBI group than in the HC group, whereas the GA genotype of SNP rs5743708 was associated with PTB (OR = 6.087; 95% CI: 1.687–21.968). The frequencies of the GG genotype of SNP rs7873784 in *TLR4* (OR = 2.136; 95% CI: 1.312–3.478) and the CC genotype of rs3764879 in *TLR8* (OR = 1.982; 95% CI: 1.292-3.042) were also significantly higher in the PTB group than in the HC group. The TC genotype frequency of SNP rs5743836 in *TLR9* was significantly higher in the LTBI group than in the HC group (OR = 1.664; 95% CI: 1.201–2.306). An MDR analysis of gene–gene and gene–environment interactions identified three SNPs (rs10759932, rs7873784, and rs10759931) that predicted LTBI with 84% accuracy (*p* = 0.0004) and three SNPs (rs3804100, rs1898830, and rs10759931) that predicted PTB with 80% accuracy (*p* = 0.0001).

**Conclusions:**

Our results suggest that genetic variation in TLR2, 4, 8 and 9, implicating TLR-related pathways affecting the innate immunity response, modulate LTBI and PTB susceptibility in Chinese.

**Electronic supplementary material:**

The online version of this article (doi:10.1186/s12881-015-0166-1) contains supplementary material, which is available to authorized users.

## Background

Mycobacterial infections remain a leading global health threat and are of major concern worldwide. The World Health Organization estimates that about one third of the world’s population is infected with *Mycobacterium tuberculosis* (Mtb), and that about 1.4 million deaths from tuberculosis (TB) occur each year [1]. Approximately 10% of individuals infected with Mtb develop active pulmonary disease, suggesting that there are differences in the susceptibility or resistance to disease development among individuals [2]. The results of twin [3] and family-based studies [4] support the role of genetics in the development of pulmonary TB (PTB). Furthermore, both human and mouse studies of mycobacterial infections have identified several potential TB-susceptibility or -resistance loci, including genes involved in toll-like receptor (TLR) signaling [5].

Bacterial infections typically result in the activation of the innate immune system as a first-line host defense mechanism. In humans, the TLRs contribute to this innate immune recognition of pathogens and shape the development of the adaptive immune response [5]. Mtb is initially recognized by TLR1, TLR2, TLR4, and TLR6, which then interact with the adaptor proteins MyD88 and toll-interleukin 1 receptor (TIR) domain containing adaptor protein (TIRAP) to activate macrophages and dendritic cells [6,7]. TLRs play an integral role in the activation of the inflammatory cytokine signaling pathways and the adaptive immune response and as a result, have become biologically plausible candidate genes in studies of TB susceptibility [8]. Human genetic studies have also indicated that variants of the TLR pathway genes, including *TLR1* [9], *TLR2* [10,11], *TLR4* [12], and *TIRAP* [13], regulate the cellular immune response and may influence human susceptibility to Mtb in different populations [14]. However, these findings have not been replicated in different populations [15], and this lack of information has hindered the discovery of genetic susceptibility factors for this disease. Therefore, it is essential that we investigate whether polymorphic variants of the TLRs are associated with susceptibility to TB in different populations, especially in areas with a high TB burden.

In China, as in the rest of the world, TB is a significant public health problem. In Shanghai, TB has a prevalence of about 7,000 newly reported cases each year [16]. In a previous study, we reported that more than 30% of latent tuberculosis infections (LTBIs) cannot be explained by socio-demographic or clinical factors, which suggests a role for genetic factors [17]. The identification of high-risk individuals among recently exposed/infected individuals is extremely important in TB control programs established to reduce the disease burden in communities. There is strong functional evidence of a genetic component involving the TLRs in human susceptibility to TB. Therefore, in the present study, we examined PTB patients, LTBI subjects, and healthy controls (HC) from the Han population in Shanghai to test the association between 16 single-nucleotide polymorphisms (SNPs) of *TLR1*, *TLR2*, *TLR4*, *TLR6*, and *TLR9* and TB, to determine the possible association of these polymorphisms with disease susceptibility. We also investigated whether potential single- or multi-locus gene interactions or gene–environment interactions can predict the susceptibility to LTBI or PTB.

## Methods

### Subjects

The patients with PTB (*n* = 109), subjects with LTBI (*n* = 225), and HCs (*n* = 422) were recruited in the metropolitan area of Shanghai, one of the largest cities in China. The incidence rate of TB in Shanghai in 2008 was 24.6 per 100,000 inhabitants, according to the municipal Center for Disease Prevention and Control (CDC).

Between 2011 and 2012, a total of 109 PTB patients were recruited from seven district CDCs, the facilities responsible for TB case management, in Shanghai. The patients included in this study were newly diagnosed with TB from sputum smear examinations for acid-fast bacilli and/or the culture of Mtb in the hospitals designated to treat TB in these districts. The exclusion criteria for patients included a positive serological test for human immunodeficiency virus (HIV) infection, organ transplantation, primary immunodeficiency, cancer, and treatment with immunosuppressive drugs, endocrine disorders such as diabetes, autoimmune or chronic renal disease, and pleural, miliary, or meningeal TB.

In total, 225 LTBI subjects and 422 healthy individuals were matched to the PTB patients by age, sex, and residency in the same district, were used as the control subjects in the present study. The individuals with LTBI were identified from the contacts of the participating PTB patients at the time of their diagnosis, and were defined as individuals who had had prolonged, frequent, or intense contact with a registered TB patient while he or she was infectious. The infectious period was defined based on the U.S. CDC guidelines [18]. The individuals with LTBI were ≥ 18 years old, HIV-seronegative, and had a positive TSPOT.*TB* result, with no evidence of active tuberculosis. The HCs were required to have a negative TSPOT.*TB* result, with no evidence of active tuberculosis. The specific criteria for the enrollment of the controls were the absence of pulmonary lesions on chest radiography and no history of TB disease. The exclusion criteria for HCs were the presence of a productive cough for more than 2 weeks, a previous history of TB, age < 15 years, and the presence of any clinical TB symptoms during the 2 years of clinical follow-up.

This study was approved by the Institutional Review Board of Fudan School of Public Health, Shanghai, and written informed consent was obtained from all the participants before blood sampling and a questionnaire-based interview.

### Determination of sample size

The sample size and statistical power were calculated with the PASS software (version 12.0; NCSS LLS, Kaysville, UT), based on the following parameters: 80% power, α = 0.05, a polymorphism prevalence of 10%, an allelic odds ratio for TB/LTBI of 2 compared with the control, and a match ratio of 2:1. Using these parameters and assumptions, the minimum sample size for each group was estimated to be 100 for the PTB group, 200 for the LTBI group, and 400 for the HC group.

### Study procedures

At the time of recruitment, the contacts of the TB patients were interviewed by trained community health workers and immediately afterwards, a 5-ml blood sample was taken from each for the TSPOT.*TB* assay. Individuals with TB symptoms or with a positive TSPOT.*TB* result were examined for TB disease with a radiological examination. The contacts were asked for their sociodemographic and clinical information. Vaccination with the bacillus Calmette–Guérin (BCG) vaccine was verified by the interviewer, who confirmed the presence of BCG scars. All data were recorded on a standardized questionnaire by the trained health workers in the district CDCs.

### Blood samples and DNA isolation

We collected 5 ml of peripheral blood from each participant into a glass tube containing potassium ethylenediaminetetraacetate. Genomic DNA was extracted with the salting-out protocol, using ammonium acetate [19]. The optical density of the DNA was measured on a Nanodrop spectrophotometer and 25–50 ng of DNA was used for each PCR.

### Genotyping

A PCR-based restriction fragment length polymorphism (PCR–RFLP) analysis was performed for *TLR1* (SNP rs5743618), *TLR2* (rs3804099, rs5743708, rs3804100, and rs1898830), *TLR4* (rs4986790, rs4986791, rs11536889, rs10759932, rs7873784, and rs10759931), *TLR6* (rs5743810), *TLR8* (129) (rs3764879), *TLR9* (rs5743836 and rs1870884), and *TIRAP* (rs8177374) using genomic DNA isolated from peripheral blood leucocytes. The forward (F) and reverse (R) primer sequences (Sungon, Shanghai, China) (Additional file 1: Table S1), and restriction enzymes (New England Biolabs, Inc., Ipswich, MA) were used to detect the restriction digestion patterns for the different alleles [13,20,21]. The PCRs were performed under the appropriate cycling conditions: initial denaturation at 95°C for 5 min, followed by 35 cycles of denaturation at 95°C for 30 s, annealing at the appropriate temperature for 30s, and extension at 72°C for 30 s, with a final extension at 72°C for 2 min. The annealing temperatures used were as follows: *TLR1*_1805: rs5743618 55°C; *TLR2*: rs3804099 65°C, rs5743708 65°C, rs3804100 63°C, and rs1898830 63°C; *TLR4*: rs4986790 56°C, rs4986791 56°C, rs11536889 54°C, rs10759932 54°C, rs7873784 55°C, and rs10759931 54°C; *TLR6*: rs5743810 52°C; *TLR8*_129: rs3764879 57°C; *TLR9*: rs5743836 59°C and rs1870884 60°C; and *TIRAP*: rs8177374 62°C. The PCR products were digested with the restriction endonucleases and the resulting fragments were separated by electrophoresis at 100 V on 3% agarose gel containing 0.5 mg/ml ethidium bromide, and visualized under UV light.

### Statistical analysis

The data were double entered on a spreadsheet (Microsoft Excel) and any discrepancies were checked with the original questionnaire data to ensure data consistency. The clinical and demographic characteristics were compared among the three groups (PTB, LTBI, and HC) with ANOVA for continuous variables and with the χ^2^ test or Fisher’s exact test for categorical variables. *p* < 0.05 was considered significant.

The allele and genotype frequencies of each polymorphism were determined by direct counting. The genotype distributions for each polymorphism were then tested for Hardy–Weinberg equilibrium values with the χ^2^ test. The genotype and allele frequencies of the different groups were compared by calculating the odds ratios and 95% confidence intervals (CI) in a conditional logistic regression model (STATA version 9.0; College Station, TX). The linkage disequilibrium (LD) coefficients *D’* and *r*^*2*^ were then calculated for the multi-locus polymorphisms studied in *TLR2* and *TLR4* to determine any co-segregation. The associations between the haplotypes and LTBI or TB were tested by calculating the logistic regression (adjustments) statistic and the corresponding *p* values and odds ratios (ORs) with 95% confidence intervals (CIs) using the SNPStats software (http://bioinfo.iconcologia.net/SNPstats/) [22]. The relationships between the *TLR* polymorphisms and the risk of PTB or LTBI were evaluated with the nonparametric MDR method [23]. Each best model was tested for its accuracy, cross-validation consistency, and significance level, determined with permutation testing, testing accuracy, and testing OR (95% CI) in the MDR analysis. Cross-validation consistency was defined as the number of cross-validation replicates (partitions) in which the same *n*-locus model was chosen as the best model (i.e., the number of replicates in which the classification error was minimized). Bonferroni corrections were applied to multiple comparisons. The level of significant was p < 0.003125 (0.05/16).

## Results

### Demographic data of the participants

The baseline characteristics of the study populations are summarized in Table 1. The age and sex distributions were very similar in all three groups. There was a significant difference between the PTB group and the LTBI group in the proportion of participants who had undergone BCG vaccination (87.2% versus 95.1%, respectively; *p* = 0.01).

**Table 1:** **Characteristics of the study participants**

**Characteristics**	**TB (** ***n*** **= 109) No. (%)**	**LTBI (** ***n*** **= 225) No. (%)**	**HC (** ***n*** **= 422) No. (%)**	**χ** ^**2**^ **/ANOVA TB.vs.LTBI**	**χ** ^**2**^ **/ANOVA LTBI.vs.HC**
Age, mean ± SD, (yrs)	45 ± 12.6	45 ± 16.5	45 ± 18.7	0.99	0.96
15~	16(14.7)	34(15.1)	63(14.9)	1.00	1.00
30~	27(24.8)	56(24.9)	105(24.9)		
45~	45(41.2)	92(40.9)	173(41.0))		
60~	21(19.3)	43(19.1)	81(19.2)		
Male	74(67.9)	152(67.6)	285(67.5)	0.951	1.000
BCG vaccination	95(87.2)	214(95.1)	405(96.0)	0.01^a^	0.99

LTBI: latent tuberculosis infection; HC, health controls. SD, standard deviation.

^a^ *p* < 0.05.

### SNP analysis

The genotype frequency distributions for all 16 SNPs investigated were consistent with Hardy–Weinberg equilibrium in all three groups, except SNPs rs3804100 (*p* = 0.012) and rs5743836 (*p* = 0.023) in the LTBI group, and rs7873784 in the LTBI (*p* = 0.0328) and PTB (*p* = 0.0375) groups.

The genotype and allele distributions were first compared between the HC and PTB groups. Compared with the HC group, the PTB group had significantly higher proportions of the GA genotype (*p* = 0.002; OR = 6.087; 95% CI: 1.687–21.968) and G allele (*p* = 0.002; OR = 5.943; 95% CI: 1.662–21.250) in rs5743708; the T allele in rs4986791 (*p* = 0.002; OR = 1.910; 95% CI: 1.260–2.896); the GG genotype in rs7873784 (*p* = 0.002; OR = 2.136; 95% CI: 1.312–3.478); the CC genotype in *TLR8*_129 (*p* = 0.002; OR = 1.982; 95% CI: 1.292–3.042); and the T allele in rs8177374 (*p* < 0.001; OR = 1.942; 95% CI: 1.238–3.048).

When we compared the genotype and allele distributions between the HC and LTBI groups, we found that the following genotypes and alleles showed significant associations with LTBI: rs3804100 (CC, *p* < 0.001; OR = 2.262; 95% CI = 1.433–3.570; C, *p* < 0.001; OR = 1.566; 95% CI = 1.233–1.990) and rs5743836 (TC, *p* = 0.002; OR = 1.664; 95% CI = 1.201–2.306).

There were no significant differences in the genotype or allele frequencies between the PTB patients and LTBI subjects. No other SNPs differed significantly in their genotypes (Table 2) or allele frequencies (Table 3) between the PTB patients, the LTBI subjects, and the HCs.

**Table 2:** **Distributions of toll-like receptor genotypes in the three groups**

**dbSNPS**	**Genotypes**	**Frequencies (No., %)**				**LTBI.vs.HC**			**TB.vs.LTBI**			**TB.vs.HC**	
		**HC (** ***n*** **= 422)**	**LTBI (** ***n*** **= 225)**	**TB (** ***n*** **= 109)**	***p***	**OR**	**95% CI**	***p***	**OR**	**95% CI**	***p***	**OR**	**95% CI**
rs5743618	GG	2(0.5)	2(0.9)	1(0.9)	0.613	1.883	0.264-13.461	0.979	1.032	0.093-11.511	0.499	1.944	0.175-21.64
	TG	70(16.6)	23(10.2)	10(9.2)	0.028	0.573	0.347-0.946	0.763	0.887	0.407-1.936	0.054	0.508	0.252-1.022
	TT	350(82.9)	200(88.9)	98(89.9)	0.043	1.646	1.011-2.678	0.778	1.114	0.526-2.356	0.074	1.833	0.935-3.592
rs3804099	CC	51(12.1)	25(11.1)	9(8.3)	0.714	0.909	0.547-1.512	0.419	0.720	0.324-1.600	0.260	0.655	0.312-1.375
	TC	180(42.6)	95(42.2)	36(33.0)	0.916	0.982	0.708-1.363	0.107	0.675	0.418-1.089	0.068	0.663	0.426-1.033
	TT	191(45.3)	105(46.7)	64(58.7)	0.732	1.058	0.765-1.464	0.039	1.625	1.023-2.581	0.012	1.720	1.123-2.636
rs5743708	GA	4(0.9)	9(4.0)	6(5.5)	0.008	4.354	1.326-14.301	0.534	1.398	0.485-4.033	0.002	6.087	1.687-21.968^a^
	GG	418(99.1)	216(96.0)	103(94.5)	0.008	0.229	0.051-0.836	0.534	0.715	0.221-2.513	0.002	0.164	0.046-0.593^a^
rs3804100	CC	42(10.0)	45(20.0)	21(19.2)	<0.001	2.262	1.433-3.570^a^	0.875	0.955	0.536-1.700	0.007	2.159	1.218-3.829
	TC	168(39.8)	90(40.0)	44(40.4)	0.963	1.008	0.724-1.402	0.949	1.015	0.637-1.619	0.916	1.023	0.666-1.572
	TT	212(50.2)	90(40.0)	44(40.4)	0.013	0.660	0.476-0.917	0.949	0.985	0.618-1.570	0.066	0.671	0.437-1.028
rs1898830	AA	142(33.6)	68(30.2)	33(30.3)	0.375	0.854	0.602-1.211	0.992	1.003	0.609-1.649	0.504	0.856	0.543-1.351
	AG	196(46.4)	113(50.2)	54(49.5)	0.360	1.163	0.842-1.608	0.907	0.973	0.616-1.538	0.564	1.132	0.743-1.725
	GG	84(20.0)	44(19.6)	22(20.2)	0.915	0.978	0.651-1.469	0.893	1.040	0.587-1.843	0.948	1.018	0.602-1.720
rs4986790	AA	346(82.0)	181(80.4)	77(70.6)	0.630	0.904	0.598-1.365	0.045	0.585	0.345-0.991	0.009	0.529	0.327-0.855
	AG	75(17.8)	42(18.7)	31(28.4)	0.778	1.062	0.699-1.613	0.043	1.732	1.094-3.050	0.013	1.839	1.132-2.987
	GG	1(0.2)	2(0.9)	1(0.9)	0.245	3.776	0.341-41.869	0.979	1.032	0.093-11.51	0.369	3.898	0.242-62.825
rs4986791	CC	342(81.0)	179(79.6)	74(67.9)	0.649	0.910	0.607-1.365	0.020	0.543	0.324-0.911	0.003	0.495	0.309-0.791
	CT	76(18.0)	43(19.1)	32(29.4)	0.730	1.076	0.710-1.629	0.035	1.759	1.036-2.987	0.009	1.892	1.169-3.061
	TT	4(1.0)	3(1.3)	3(2.7)	0.699	1.412	0.313-6.365	0.396	2.094	0.416-10.551	0.141	2.958	0.652-13.415
rs11536889	CC	34(8.1)	17(7.6)	10(9.2)	0.822	0.933	0.509-1.701	0.611	1.236	0.546-2.798	0.706	1.153	0.551-2.413
	GC	167(39.5)	86(38.2)	40(36.7)	0.737	0.945	0.678-1.317	0.787	0.937	0.584-1.504	0.583	0.885	0.573-1.368
	GG	221(52.4)	122(54.2)	59(54.1)	0.653	1.077	0.779-1.490	0.987	0.996	0.630-1.577	0.743	1.073	0.703-1.637
rs10759932	CC	42(10.0)	21(9.3)	5(4.5)	0.800	0.931	0.537-1.616	0.129	0.467	0.171-1.274	0.079	0.435	0.168-1.127
	TC	168(39.8)	90(40.0)	49(45.0)	0.963	1.008	0.724-1.402	0.389	1.225	0.772-1.945	0.330	1.235	0.807-1.888
	TT	212(50.2)	114(50.7)	55(50.5)	0.917	1.017	0.736-1.406	0.972	0.992	0.628-1.567	0.967	1.009	0.662-1.537
rs7873784	CC	29(6.9)	19(8.4)	7(6.4)	0.467	1.250	0.684-2.283	0.518	0.744	0.303-1.827	0.868	0.930	0.396-2.184
	GC	135(32.0)	57(25.4)	18(16.5)	0.078	0.721	0.501-1.037	0.070	0.583	0.324-1.050	0.001	0.421	0.244-0.726^a^
	GG	258(61.1)	149(66.2)	84(77.1)	0.202	1.246	0.888-1.748	0.043	1.714	1.014-2.897	0.002	2.136	1.312-3.478^a^
rs10759931	AA	143(33.9)	90(40.0)	44(40.4)	0.123	1.301	0.931-1.817	0.949	1.015	0.637-1.619	0.207	1.321	0.857-2.035
	AG	215(50.9)	113(50.2)	54(49.5)	0.860	0.971	0.703-1.343	0.907	0.973	0.616-1.538	0.793	0.945	0.620-1.440
	GG	64(15.2)	22(9.8)	11(10.1)	0.055	0.606	0.363-1.014	0.928	1.036	0.483-2.221	0.175	0.628	0.319-1.236
rs5743810	CT	12(2.8)	8(3.6)	5(4.6)	0.618	1.260	0.507-3.128	0.648	1.304	0.416-4.084	0.357	1.643	0.566-4.766
	TT	410(97.2)	217(96.4)	104(95.4)	0.618	0.790	0.293-2.276	0.648	0.773	0.215-3.057	0.357	0.609	0.210-1.766
TLR8_129	CC	133(31.5)	81(36.0)	52(47.7)	0.248	1.222	0.869-1.719	0.040	1.622	1.020-2.580	0.002	1.982	1.292-3.042^a^
	CG	190(45.0)	96(42.7)	41(37.6)	0.565	0.909	0.656-1.259	0.379	0.810	0.507-1.295	0.164	0.736	0.478-1.134
	GG	99(23.5)	48(21.3)	16(14.7)	0.539	0.885	0.599-1.307	0.147	0.634	0.342-1.178	0.047	0.561	0.315-0.999
rs5743836	CC	25(5.9)	13(5.8)	6(5.5)	0.940	0.974	0.488-1.943	0.919	0.950	0.351-2.571	0.868	0.925	0.370-2.314
	TC	181(42.9)	125(55.5)	49(45.0)	0.002	1.664	1.201-2.306	0.069	0.653	0.412-1.035	0.698	1.087	0.712-1.661
	TT	216(51.2)	87(38.7)	54(49.5)	0.002	0.601	0.433-0.836	0.059	1.557	0.982-2.471	0.760	0.936	0.614-1.427
rs1870884	CC	3(0.7)	1(0.4)	0(0)	1.000	0.624	0.064-6.029		-				
	TC	107(25.4)	56(24.9)	27(24.8)	0.896	0.976	0.672-1.417	0.981	0.994	0.585-1.687	0.900	0.969	0.596-1.578
	TT	312(73.9)	168(74.7)	82(75.2)	0.839	1.039	0.717-1.506	0.912	1.030	0.607-1.748	0.783	1.071	0.658-1.741
TIRAP_975	CC	313(74.2)	153(68.0)	61(56.0)	0.096	0.740	0.519-1.055	0.032	0.598	0.374-0.957	<0.001	0.443	0.286-0.685^a^
	CT	97(23.0)	61(27.1)	40(36.7)	0.245	1.246	0.860-1.806	0.074	1.559	0.957-2.539	0.004	1.942	1.238-3.048
	TT	12(2.8)	11(4.9)	8(7.3)	0.181	1.756	0.762-4.046	0.365	1.541	0.601-3.949	0.028	2.706	1.078-6.796

PTB, pulmonary tuberculosis; LTBI, latent tuberculosis infection; HC, healthy control.

^a^ *p* < 0.003125 (0.05/16), the difference was statistically significant after correction for multiple testing (Bonferroni correction).

**Table 3:** **Distribution of toll-like receptor alleles in the three groups**

**dbSNPS**		**Frequencies (No., %)**			**LTBI.vs.HC**			**TB.vs.LTBI**			**TB.vs.HC**		
	**Alleles**	**HC (** ***n*** **= 422)**	**LTBI (** ***n*** **= 225)**	**TB (** ***n*** **= 109)**	***p***	**OR**	**95% CI**	***p***	**OR**	**95% CI**	***p***	**OR**	**95% CI**
TLR1_1805	G	74(8.7)	27(6.0)	12(5.5)	0.077	0.664	0.404-1.064	0.798	0.913	0.412-1.908	0.115	0.606	0.323-1.137
	T	770(91.3)	423(94.0)	206(94.5)	0.077	1.506	0.940-2.473	0.798	1.095	0.524-2.425	0.115	1.650	0.880-3.095
rs3804099	C	282(33.4)	145(32.2)	54(24.8)	0.665	0.947	0.736-1.217	0.048	0.693	0.471-1.012	0.014	0.656	0.467-0.921
	T	562(66.6)	305(67.8)	164(75.2)	0.665	1.055	0.859-1.244	0.048	1.443	0.988-2.124	0.014	1.524	1.086-2.139
rs5743708	A	840(99.5)	441(98.0)	212(96.8)	0.009	0.233	0.052-0.843	0.538	0.721	0.226-2.500	0.002	0.168	0.047-0.602^a^
	G	4(0.5)	9(2.0)	6(3.2)	0.009	4.291	1.187-19.13	0.538	1.386	0.401-4.429	0.002	5.943	1.662-21.25^a^
rs3804100	C	252(29.9)	180(40.0)	86(39.4)	<0.001	1.566	1.223-2.003^a^	0.892	0.977	0.692-1.378	0.007	1.531	1.124-2.085
	T	592(70.1)	270(60.0)	132(60.0)	<0.001	0.638	0.499-0.818^a^	0.892	1.023	0.742-1.387	0.007	0.653	0.480-0.890
rs1898830	A	480(56.9)	249(55.3)	120(55.0)	0.595	0.939	0.741-1/191	0.944	0.988	0.705-1.387	0.628	0.929	0.688-1.253
	G	364(43.1)	201(44.7)	98(45.0)	0.595	1.064	0.840-1.349	0.944	1.012	0.721-1.419	0.628	1.077	0.798-1.453
rs4986790	A	767(90.9)	404(89.8)	185(84.9)	0.521	0.882	0.591-1.325	0.065	0.638	0.385-1.068	0.009	0.563	0.363-0.872
	G	77(9.1)	46(10.2)	33(15.1)	0.521	1.133	0.754-1.691	0.065	1.567	0.937-2.595	0.009	1.777	1.146-2.754
rs4986791	C	760(90.0)	401(89.1)	180(82.6)	0.597	1.104	0.690-1.556	0.018	0.579	0.309-0.838^a^	0.002	0.524	0.345-0.794^a^
	T	84(10.0)	49(10.9)	38(17.4)	0.597	0.905	0.642-1.449	0.018	1.727	1.193-3.235^a^	0.002	1.910	1.260-2.896^a^
rs11536889	C	235(27.8)	120(26.7)	60(72.5)	0.651	0.942	0.721-1.228	0.815	1.044	0.712-1.523	0.925	0.984	0.705-1.373
	G	609(72.2)	330(73.3)	158(27.5)	0.651	1.061	0.814-1.386	0.815	0.958	0.657-1.404	0.925	1.016	0.728-1.418
rs10759932	C	252(29.9)	132(29.3)	59(27.1)	0.844	0.975	0.752-1.262	0.543	0.894	0.611-1.300	0.419	0.872	0.625-1.216
	T	592(70.1)	318(70.7)	159(72.9)	0.844	1.026	0.792-1.330	0.543	1.119	0.769-1.637	0.419	1.147	0.822-1.601
rs7873784	C	193(22.9)	95(21.1)	32(14.7)	0.469	0.903	0.713-1.270	0.047	0.643	0.401-0.912	0.008	1.632	1.084-2.456
	G	651(77.1)	355(78.9)	186(85.3)	0.469	1.107	0.787-1.403	0.047	1.555	1.017-2.494	0.008	0.613	0.407-0.923
rs10759931	A	501(59.4)	293(65.1)	142(65.1)	0.043	1.278	0.910-1.488	0.995	1.001	0.704-1.429	0.120	1.164	0.853-10589
	G	343(40.6)	157(34.9)	76(34.9)	0.043	0.782	0.672-1.099	0.995	0.999	0.700-1.421	0.120	0.859	0.629-1.173
rs5743810	C	12(1.4)	8(1.8)	5(2.3)	0.621	1.255	0.441-3.366	0.710	1.238	0.330-4.557	0.361	1.628	0.567-4.670
	T	832(98.6)	442(98.2)	213(97.7)	0.621	0.197	0.297-2.265	0.710	0.807	0.219-3.034	0.361	0.614	0.214-1.763
TLR_129	C	456(54.0)	258(57.3)	145(66.5)	0.255	1.143	0.774-1.251	0.023	1.478	0.674-1.335	0.001	0.932	0.689-1.261^a^
	G	388(46.0)	192(42.7)	73(33.5)	0.255	0.875	0.799-1.292	0.023	0.676	0.749-1.484	0.001	1.073	0.793-1.451^a^
rs5743836	C	231(27.4)	151(35.6)	61(28.0)	0.020	1.340	1.038-1.728	0.147	0.769	0.530-1.111	0.857	1.031	0.740-1.437
	T	613(72.6)	299(66.4)	157(72.0)	0.020	0.746	0.579-0.964	0.147	1.300	0.900-1.888	0.857	0.970	0.696-1.352
rs1870884	C	113(13.4)	58(12.9)	27(12.4)	0.800	0.957	0.669-1.359	0.855	0.955	0.563-1.591	0.696	0.914	0.584-1.433
	T	731(86.6)	392(87.1)	191(87.6)	0.800	1.044	0.736-1.494	0.855	1.047	0.629-1.777	0.696	1.094	0.698-1.713
TIRAP_975	C	723(85.7)	367(81.6)	162(74.3)	0.053	0.740	0.539-1.019	0.031	0.654	0.438-0.983^a^	<0.001	0.484	0.338-0.694^a^
	T	121(14.3)	83(18.4)	56(25.7)	0.053	1.351	0.981-1.855	0.031	1.535	1.017-2.286^a^	<0.001	2.066	1.442-2.960^a^

PTB, pulmonary tuberculosis; LTBI, latent tuberculosis infection; HC, healthy control.

^a^ *p* < 0.003125 (0.05/16), the difference was statistically significant after correction for multiple testing (Bonferroni correction).

### Haplotype analysis

**Figure 1:**
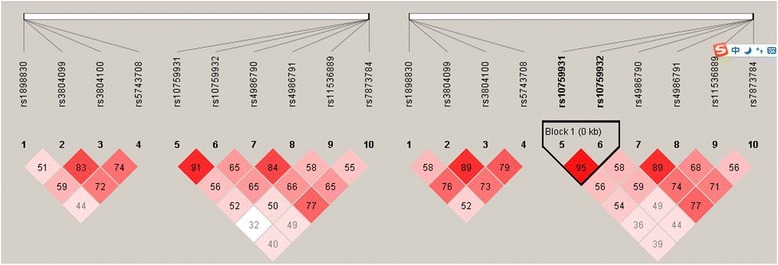
**LD block structure in TLR2 and TLR4 and in the proximity of the gene in relation to LTBI (left) and PTB (right).**

Two haplotype block sets in *TLR2* and *TLR4* were identified with the haplotype analysis (Table 4). We selected four polymorphisms in *TLR2* and eight polymorphisms in *TLR4* after setting the threshold for the LD coefficient (*D’* > 0.80; Figure 1). The haplotype frequencies of *TLR4* were significantly higher in the PTB group than in the LTBI group, for rs10759931/rs10759932 (AT, *p* < 0.001, OR = 2.00, 95% CI = 1.221–3.289) and rs4986790/rs4986791 (AT, *p* < 0.001, OR = 3.59, 95% CI = 1.570–8.565). However, the frequencies of the other haplotypes of *TLR2* and *TLR4* did not differ significantly among the three groups.

**Table 4:** **Haplotypes of the** ***TLR2*** **and** ***TLR4*** **genes and their distributions in the three groups**

**SNP marker**	**Haplotype**	**Frequency (No., %)**			**LTBI.vs.HC**			**PTB.vs.LTBI**		
		**HC (** ***n*** **= 422)**	**LTBI (** ***n*** **= 225)**	**PTB (** ***n*** **= 109)**	***P***	**OR**	**95% CI**	***P***	**OR**	**95% CI**
rs3804099	CC	183(21.7)	95(21.1)	52(23.8)	0.812	0.97	0.722-1.289	0.422	1.17	0.779-1.747
rs3804100	CT	185(21.9)	85(18.9)	36(16.5)	0.201	0.83	0.615-1.114	0.455	0.85	0.537-1.326
	TC	103(12.2)	56(12.4)	18(8.3)	0.900	1.02	0.709-1.465	0.106	0.63	0.341-1.129
	TT	373(44.2)	214(47.6)	112(51.4)	0.247	1.15	0.904-1.450	0.354	1.17	0.832-1.632
rs10759931	AT	376(44.4)	204(45.3)	136(62.4)	0.787	1.03	0.735-1.447	<0.001^a^	2.00	1.221-3.289
rs10759932	GT	218(25.7)	112(24.9)	22(10.1)	0.712	0.95	0.642-1.401	<0.001^a^	0.34	0.153-0.694
	GC	156(18.5)	88(19.6)	6(2.8)	0.639	1.07	0.693-1.646	<0.001^a^	0.12	0.022-0.379
	AC	94(11.4)	46(10.2)	54(24.7)	0.614	0.91	0.511-1.578	<0.001^a^	2.89	1.496-5.596
rs4986790	AC	644(76.3)	353(78.4)	157(72.0)	0.383	1.13	0.852-1.505	0.067	0.71	0.481-1.046
rs4986791	AT	49(5.8)	11(2.4)	18(8.3)	0.006	0.39	0.180-0.784	0.001^a^	3.59	1.570-8.565
	GC	33(3.9)	9(2.0)	0(0)	0.065	0.50	0.209-1.083	-	-	-
	GT	118(14.0)	77(17.1)	43(19.7)	0.134	1.27	0.915-1.755	0.409	1.19	0.766-1.833

PTB, pulmonary tuberculosis; LTBI, latent tuberculosis infection; HC, healthy control.

^a^ *p* < 0.003125 (0.05/16), the difference was statistically significant after correction for multiple testing (Bonferroni correction).

### Gene–gene and gene–environment interactions evaluated with nonparametric MDR

After cross-validation and permutation tests of the gene–gene and gene–environment interactions in relation to the LTBI group, the best models included a one-marker model (rs5743836) with 56% balanced accuracy and 9/10 cross-validation consistency, a two-marker model (rs3804100, rs1898830) with 69% balanced accuracy and 10/10 cross-validation consistency, and a three-marker model (rs3804100, rs1898830, rs10759931) with 80% balanced accuracy and 10/10 cross-validation consistency (Table 5). The interaction dendrogram also indicated a synergistic effect between rs3804100 and rs10759931.

**Table 5:** **MDR analysis of gene–gene and gene–environment interactions in relation to LTBI and PTB**

**Best model**	**Testing accuracy**	**Testing sensitivity**	**Testing OR**	**Testing χ2**	***p***	**Cross-validation consistency**
PTB.vs.LTBI						
BCG	0.61	0.70	1.76(1.067-2.903)^a^	4.958	0.026^a^	6/10
rs10759932, rs7873784	0.76	0.72	3.29(2.096-8.283)^a^	9.253	0.0024^a^	10/10
rs10759932, rs7873784, rs10759931	0.84	0.81	5.67(3.158-14.99)^a^	12.67	0.0004^a^	10/10
LTBI.vs.HC						
rs5743836	0.56	0.55	1.68(1.189-2.365)^a^	8.76	0.0031^a^	9/10
rs3804100, rs1898830	0.69	0.63	4.57(1.532-13.61)^a^	7.88	0.005^a^	10/10
rs3804100, rs1898830, rs10759931	0.80	0.79	5.76(3.332-16.07)^a^	21.85	0.0001^a^	10/10

PTB, pulmonary tuberculosis; LTBI, latent tuberculosis infection; HC, healthy control;MDR, multifactor dimensionality reduction.

^a^ *p* < 0.05.

Regarding the role of these interactions in predicting PTB, we identified a one-marker model (BCG vaccination) that had maximum cross-validation consistency and a maximum prediction accuracy of 61%, a two-marker model (rs10759932, rs7873784) with 76% balanced accuracy and a cross-validation consistency of 100% in predicting PTB risk (*p* = 0.0024 based on 1000-fold permutation testing), and a three-marker model (rs10759932, rs7873784, rs10759931) with 84% balanced accuracy and a cross-validation consistency of 100% (Table 5). The interaction dendrogram shows the amount of information obtained about LTBI versus HC using MDR, and indicates a synergistic effect between rs10759932 and rs7873784.

## Discussion

Shanghai is a city with a moderate incidence of TB, reaching 33.7 per 100,000 population in 1999 and 26.3 per 100,000 in 2000. Under these circumstances, all children born in Shanghai are routinely vaccinated with the BCG vaccine soon after birth. Therefore, the tuberculin skin test has a high positive rate in China, but a positive tuberculin skin test does not conclusively distinguish between exposure *via* contact with Mtb and exposure *via* BCG vaccination. Therefore, in this study, we used the TSPOT.*TB* test to differentiate between LTBI subjects and individuals who were not infected with Mtb.

We examined 16 markers in seven TLR-related genes for their association with PTB or LTBI. These six *TLRs* were selected because there is strong biological evidence of their roles in disease susceptibility. We observed statistically significant associations between *TLR2*, *TLR4*, and *TLR9* and susceptibility to PTB or LTBI.

The essential role of TLR2 against mycobacterial infection has been demonstrated *in vivo* by the rapid death [24,25] and higher Mtb burden in TLR2-deficient mice [25]. Therefore, it is reasonable to suggest that a subtle reduction in the expression of TLR2 could also make human more susceptible to the development of TB. In this study, the genotype and allele distributions of *TLR2* (rs3804100) differed significantly between the LTBI and HC groups, and this may be the first report of an association between rs3804100 and LTBI in China. These data suggest that a defective *TLR2* gene is a causative factor for increased susceptibility to LTBI and its subsequent progression to PTB disease. Therefore, the detection of this polymorphism among TB patients may provide important information in the assessment of their risk profiles for susceptibility to TB. We found that the Arg753Gln (rs5743708) polymorphism was a risk factor for TB in our Chinese population, which is slightly different from the results of other studies of Vietnamese patients with TB meningitis [26], Turkish patients with TB [11,27], and a Croatian Caucasian population [10]. This discrepancy may result from differences in the TB diagnostic criteria used, the genetics of the populations studied, or differences in sample sizes or analytic approaches used.

Several investigators have studied the roles of *TLR4* genetic polymorphisms in major infectious diseases, including TB. In the present study, the rs7873784 polymorphism showed a strong association with PTB in our Chinese population. As reported previously in studies of Chinese [28], Indonesian [29], and Vietnamese populations [30], the 299Gly mutation was almost absent in the present study. However, the AG genotype of rs4986791 was related to PTB disease. This genetic variation in rs4986791 could alter the extracellular domain of the protein, which may modulate the interaction of ligands, such as lipopolysaccharide, with TLR4 [31], leading to an impaired immune response and aggravated infection. The LD observed between the two *TLR4* variants (rs4986790 and rs4986791) in the HC population (*D’* = 0.82) was also relatively weaker than the LD reported among European, Japanese, and other populations (*D’* > 0.9). This discrepancy could result from different environmental and pathogen-induced selection pressures, causing race-specific differences in the patterns of evolutionary distribution. The two cosegregating mutations, Thr399Ile and Asp299Gly, which occur in the ectoplasmic leucine-rich repeat domain of TLR4, are significantly associated with a reduced cytokine response to lipopolysaccharide stimulation [32] and increased susceptibility to a variety of infections [33,34] by affecting the extracellular domain of TLR4 [35]. We also found that SNP rs7873784, in the 3′-untranslated region (3UTR) of *TLR4*, was significantly associated with PTB in the study population. The effect of the rs7873784 variant on TB has not been described before. However, rs7873784 was shown to be associated with a reduced risk of prostate cancer in an American population [36]. Together with the results of the present study, these findings imply that the 3′-UTR SNP rs7873784 potentially influences the development of various diseases, making it a good candidate functional SNP.

TLR9 is also known to play an important role in the activation of the innate immune system. It is the receptor for viral and bacterial CpG DNA motifs, and several studies have shown that the binding of TLR9 is necessary to drive the Th1 immune response [37,38]. In the present study, the *TLR9* polymorphism rs5743836 was associated with the risk of LTBI. This polymorphism in *TLR9* has been consistently associated with increased transcriptional activity [39,40], supporting the notion that rs5743836 enhances TLR9 function and increases susceptibility to LTBI.

TIRAP has two isoforms, both of which have a C-terminal TIR domain that mediates signals from TLR2 and TLR4. However, in the present study, no association was observed between the TIRAP polymorphism (975C/T) Ser180Leu and PTB or LTBI.

Because TLR6 mediates the recognition of lipopeptides when it forms a heterodimer with TLR2, we also examined the *TLR6* 745C/T (rs5743810) polymorphism and observed a lower frequency of the C allele in the PTB and LTBI groups than in the HC group. However, the frequency was too low for a proper statistical evaluation, so the possible association between this *TLR6* polymorphism and human susceptibility to TB disease is yet to be confirmed in a Chinese population.

Several loci usually contribute to the phenotypes expressed in complex diseases, including TB. Therefore, it is important to identify gene–gene interactions (epistasis), because they may more accurately predict the risk of disease than single genes. In the present study, an MDR analysis was used to predict potential gene–gene and gene–environment interactions that may partly determine the complex phenotypes produced by Mtb pathogenesis. We found that rs3804100, rs1898830, and rs10759931 were associated with LTBI, whereas rs10759932, rs7873784, and rs10759931 were associated with PTB. These findings are consistent with the recently identified links between TLRs and the innate immune response to Mtb, i.e., the relationship between TLR signaling, the upregulated expression of the vitamin D receptor, and the vitamin-D-mediated killing of intracellular Mtb by the antimicrobial peptide cathelicidin [41]. However, these SNPs cannot be used to interpret the immunological findings that pertain to LTBI and subsequent PTB. Nonetheless, as noted above, they suggest that different cytokine pathways are important in LTBI and PTB. Further studies are required to determine whether these polymorphisms can account for this epidemiological finding.

The strength of this study was the differentiation of LTBI cases from HCs, which provided an opportunity to analyze the impact of TLR polymorphisms on the susceptibility to both LTBI and PTB. However, this study had some limitations. The individuals in the three groups were not matched for other risk factors (e.g., BCG vaccination), so their susceptibility to PTB and LTBI may have resulted not only from genetic factors. However, gene–gene and gene–environment interactions were evaluated in relation to LTBI and PTB. Furthermore, a few SNPs identified in the LTBI and PTB groups deviated slightly from Hardy–Weinberg equilibrium in the HC group. However, these deviations were not significant (*p* > 0.01) and did not suggest significant genotyping errors. The observed deviations from Hardy–Weinberg equilibrium in the TLR polymorphisms in this Chinese population may indicate a functional effect, suggesting that more power may be necessary to observe such associations in China. We acknowledge that an important limitation of our study is the small sample size, which may also have a significant impact on observed statistical significance. This is the case of SNP rs5743708, where homozygotes for the minor allele were not detected and minimal heterozygotes’ frequency fluctuations would obliterate the positive association result. Replication by independent studies with adequately powered sample size, will be necessary to confirm or refute our findings.

## Conclusion

Taken together, our results suggest that polymorphisms in *TLR2* influence the risk of LTBI and subsequent PTB in the Chinese population, and that variations in *TLR4* and *TLR9* influence the risk of LTBI and PTB disease, respectively. Clarification of the precise roles that these genes play in TB susceptibility will require the isolation of functional variants of TLR2, TLR4, and TLR9 that can explain these variations. Epidemiological studies and basic and genetic research will extend our understanding of the critical determinants of host susceptibility to Mtb in different populations, and may provide new insights into the effects of genetic heterogeneity on the development of the different stages of TB.

## Additional file

Additional file 1: Table S1. Primer sequences and restriction enzymes used for genotyping the studied TLR genes.

## Abbreviations

TLR: Toll-like receptor
LTBI: Latent tuberculosis infection
TB: Tuberculosis
PTB: Pulmonary tuberculosis
PCR–RFLP: Polymerase chain reaction–restriction fragment length polymorphism
MDR: Multifactor dimensionality reduction
CDC: Center for Disease Prevention and Control
