# Supplementary material for: Screening toll-like receptor markers to predict latent tuberculosis infection and subsequent tuberculosis disease in a Chinese population
Source: BMC Med Genet. 2015 Apr 1;16:19. doi: 10.1186/s12881-015-0166-1 (PMC4421918; doi:10.1186/s12881-015-0166-1)
Supplement: Additional file 1: Table S1. — Primer sequences and restriction enzymes used for genotyping the studied TLR genes. [file 12881_2015_166_MOESM1_ESM.doc]

Table S1 Primer sequences and restriction enzymes used for genotyping the studied TLR genes

|  | Sequences of the primers | Restriction enzymes used |
| --- | --- | --- |
| rs5743618 | F: 5’ GGAAAGTTATAGAGGAACCCT 3’  R: 5’ CTTCACCCAGAAAGAATCGTGCC 3’ | *AluI^[13]^* |
| rs3804099 | F: 5’TGTAAAACGACGGCCAGT 3’  R: 5’CAGGAAACAGCTATGACC 3’ | *HpyCH4^[20]^* |
| rs5743708 | F: 5’ CATTCCCCAGCGCTTCTGCAAGCTCC 3’  R: 5’GGAACCTAGGACTTTATCGCAGCTC 3’ | *MspI^[21]^* |
| rs3804100 | F: 5’GTAACAGGCTGCATTCCCAAGACAC 3’  R: 5’ACTTATCCAGCACACGAATACACAG 5’ | *NeoI^[20]^* |
| rs1898830 | F: 5’GAACAGGGGAAATAATAATATAAGA 3’  R: 5’ATAGTAAAATAAATCCAGAGAAA 3’ | *MwoI^[21]^* |
| rs4986790 | F: 5’AGCATACTTAGACTACTACCTCCATC 3’  R: 5’GGAACCTAGGACTTTATCGCAGCTC 3’ | *NcoI^[21]^* |
| rs4986791 | F: 5’ GGTTGCTGTTCTCAAAGTGATTTTGGGGAGTT 3’  R: 5’GGAAATCCAGATGTTCTAGTTGTTCTAAGCC 3’ | *HinfI^[21]^* |
| rs11536889 | F: 5’TGTAAAACGACGGCCAGT 3’  R: 3’CAGGAAACAGCTATGACC 3’ | *HinfI^[20]^* |
| rs10759932 | F: 5’GTTGGTGAAGATGTGAAAAAATGAG 3’  R: 5’TTACAGACCAGAAAGTAATAATAAG 3’ | *KpnI^[20]^* |
| rs7873784 | F: 5’TATAATGAACAATACTGTATTATGC 3’  R: 5’AAAGATCAGCTGTATAGCAGAGTTC 3’ | *XhoI^[20]^* |
| rs10759931 | F: 5’TACCTGGACCTGTGATGATTAGGGC 3’  R: 5’TAGAGGGTCTGTCTCTAGTTGTCTG 3’ | *NotI^[20]^* |
| rs5743810 | F: 5’GCATTTCCAAGTCGTTTCTATGT 3’  R: 5’GCAAAAACCCTTCACCTTGTT 3’ | *AvaII^[13]^* |
| rs3764879 | F: 5’TGTAAAACGACGGCCAGT 3’  R: 5’CAGGAAACAGCTATGACC 3’ | *BfaI^[20]^* |
| rs5743836 | F: 5’CTGCTTGCAGTTGACTGTGT 3’  R: 5’ATGGGAGCAGAGACATAATGGA 3’ | *BstNI^[13]^* |
| rs1870884 | F: 5’ TATCGTCTTATTCCCCTGCTGGAATGT 3’  R: 5’TGCCCAGAGCTGACTGCTGG 3’ | *Afl II^[13]^* |
| rs8177374 | F: 5’ TGCTCATCACGCCGGGCTTCCTT 3’  R: 5’ TAGGCAGCTCTGCTGAGGTCC 3’ | *Hpy 188I^[13]^* |
